# Obstacles to Obtaining Optimal Physiotherapy Services in a Rural Community in Southeastern Nigeria

**DOI:** 10.1155/2012/909675

**Published:** 2012-08-30

**Authors:** Chinonso Igwesi-Chidobe

**Affiliations:** Department of Medical Rehabilitation, Faculty of Health Sciences and Technology, College of Medicine, University of Nigeria, Enugu Campus, 400006 Enugu, Nigeria

## Abstract

*Background*. Many people continue to live with physical disabilities across the globe, especially in rural Africa despite expertise of Physiotherapists and available evidence of effectiveness of Physiotherapy. *Objective*. To determine the obstacles to obtaining Optimal Physiotherapy services in a rural community in Southeastern Nigeria. *Methods*. Population-based cross-sectional study of individuals and health facilities in a rural community in Southeastern Nigeria. *Results*. The obstacles to obtaining optimal physiotherapy services in this community were unavailability of physiotherapy services, poor knowledge of health workers and community dwellers of the roles and scope of physiotherapy, poor health care seeking behavior of community dwellers, patronage of traditional health workers, and poor referral practices by health workers. *Conclusion*. Rural health workers in Nkanu West Local Government and other rural communities in Nigeria and Africa should be educated on the roles and scope of physiotherapy. There is a need for raising awareness of the management options for movement/functional problems for rural indigenous communities in Nigeria in particular and Africa in general. Physiotherapists should be made aware of the growing need for physiotherapy in rural areas of Nigeria and Africa largely comprising of the elderly.

## 1. Introduction

Concomitant with the aging of our population is a significant rise in the prevalence of chronic diseases. This in turn has increased the need for physical therapists and physical therapy services by all health agencies. The unprecedented need for services may outstrip the capabilities of the existing medical facilities. There is also an increasing need for out-of-hospital treatment programs with a concurrent shortage of competent physical therapists to staff them [1].

Despite the expertise in therapeutic exercises and the available evidence of effectiveness, many people continue to live with physical disabilities across the globe, especially in Africa [2–4]. Access to rehabilitation for people with disability is inadequate, more so in rural communities, with the attendant economic and social implications if the status quo is maintained [2–4]. Webster et al. in 2008 stated that despite physiotherapy being regarded positively by all referral groups of patients, there is still a distinct lack of knowledge about the profession by the general public, which affects self-referral [4]. Different referral practices exist among doctors based on the different views held by these doctors as to the conditions considered amenable to physiotherapy or their therapeutic intentions when prescribing physiotherapy with or without other medications as discussed by Akpala et al. [5]. They stated that other factors that might influence referral patterns could be the age, sex of individual doctors, medical school attended, and previous experience of hospital or other rehabilitation services, and particularly of physical therapy [5]. There is also a problem in the employment of evidence-based practice by many physical therapists. Jette et al. noted that physical therapists had a need to increase the use of evidence in their daily practice [6]. The low concept of public-health-oriented physical therapy can be seen among several physical therapists. Raman and Levi in USA stated that many conceptualized disability as individual limitations within specific contexts and infrequently conceptualized disability as a societal phenomenon affecting persons across most settings and circumstances. They believed that a concept of disability that is more inclusive of broad, as well as specific contexts of disability may lead to improved physical therapy management for individuals with a wide range of performance capacities [3]. 

According to the chartered society of physiotherapy in 2010, physiotherapy workforce has a key role to play in the public health agenda through its contribution to the prevention of disease, promotion of good health, particularly through physical activity and improvement in the general quality of life [7]. Studies indicate the need to address these shortcomings in physiotherapy especially in the Nigerian environment [8–13]. 

Despite there being a large literature supporting the importance of physiotherapy for optimal public health of every nation's citizenry, there remains a need for a more public health-oriented evidence-based physical therapy practice [14–25]. This should be improved as well as improving other challenges of the profession like improper referral practices, suboptimal treatment choices like the use of oral NSAIDs in place of Physiotherapy, decreased awareness of others of the role and scope of the profession, poor team approach in patient management, patronage of traditional healers, and so many others [26–39].

**Significance of Work**

Majority of the populace in Nigeria reside in rural areas without any access to rehabilitation services. Furthermore these groups are often poor and marginalized with the highest level of disability and functional dependence, therefore with the highest need for rehabilitation. Unfortunately rehabilitation services are in the urban areas in Nigeria.

## 2. Materials and Method

### 2.1. Background of Study Area

Nkanu West Local Government is one of the 17 Local Government Areas (L.G.As) in Enugu State with a population of about 160,497. Enugu State is one of the five states that constitute South Eastern Nigeria with a population of 3, 257, 298 constituting 2.33% of Nigeria's total population of 140, 003, 542 people at the 2006 census. Majority of Nigeria's elderly reside in the rural areas where they engage majorly in subsistence agriculture [40]. 

The headquarters of Nkanu West are based at Agbani. The L.G.A has an area of 225 km² with 14 wards. Nkanu West Local Government is bounded on the north by Enugu South Local Government Area, on the east by Nkanu East L.G.A., on the south by Awgu Local Government Area, and on the west by Udi Local Government. The health facilities in the 14 wards in Nkanu West Local Government include those listed on Table 1.

Therefore, there was a total of 11 private health facilities, 19 primary health centers, 1 secondary health facility, 1 tertiary health facility, and 3 traditional health facilities in the entire L.G.A but the 9 wards randomly studied from the L.G.A had a total of 24 health facilities comprising 8 private health facilities, 12 primary health centers, 1 secondary health facility, 3 traditional health facilities and had no tertiary health facility. 

There are about 120 registered physiotherapists in Enugu state out of the total 2,560 registered in Nigeria.

### 2.2. Design of Study

Population-based cross-sectional study design.

### 2.3. Sampling Plan

Study population:

- household members in the communities of the study area,
- directors/Managers of health facilities in the communities.

### 2.4. Survey Instruments

- Interview of heads of households employing interviewer-administered questionnaire to assess the people's knowledge and utilization of physiotherapy services.
- Interview of the directors/managers of the 24 health facilities in the 9 chosen wards using interviewer-administered questionnaire to assess their need and knowledge of Physiotherapy: their perceived need for physiotherapy services and Physiotherapy services offered in their institution. There was also observation to assess the available manpower, equipment, and treatment protocol for physiotherapy services in each health institution by the researcher.

Three copies of these questionnaires were then sent to three experts in questionnaires design to determine their content validity. Corrections based on their input were reflected on the questionnaire before their final versions were produced. Before administration, the questionnaire, which was written in English language, was pretested by a few Physiotherapists in communities outside the study area in order to eliminate ambiguities and ensure comprehension by all respondents.

### 2.5. Sample Size Estimation, Sampling, and Data Collection

#### 2.5.1. Households

There is no reasonable estimate of the proportion of people having or lacking knowledge of physiotherapy in any community in Nigeria, hence 50% was used (0.50). Since Nkanu West Local Government has a finite population of about 160,497 people in an area of 225 km², sample size formula for proportions with population greater than 10,000 was used

(1) $$\begin{array}{c} n=\frac{z^{2}pq}{d^{2}}, \end{array}$$

where:*n* = the desired sample size (when population is greater than 10,000),  *z* = the standard normal deviate, set at 1.96 corresponding to 95% confidence level,  *p* = the proportion of persons requiring physiotherapy services, because it is not available from literature, 50% will be used (0.50),  *q* = 1.0-p,  *d* = error tolerated, set at 0.05 [14],  *n* = (1.96^2^ × 0.5 × 0.5)/(0.05)^2^ = 384  participants.

Since the questionnaires were interviewer administered, nonresponse was not anticipated hence minimum sample size was 384 participants but a total of 408 participants were studied.

Study participants in the communities were selected using a 3-stage cluster sampling method. The nine selected wards were composed of 115 villages and 12 of these villages were selected randomly. Approximately each village was located 1 km or less from the health facilities. All consenting members of alternate households in each selected village were enrolled for the study. A total of 34 participants (heads of households) were enrolled in each village making a total of 408 participants but data for 400 participants were finally analyzed. 

#### 2.5.2. Health Facilities

 Cluster sampling method was used to select health facilities. Nine wards out of the fourteen wards that make up Nkanu West Local Government Area were chosen randomly. All the health facilities in the 9 wards comprising 8 private health facilities, 12 primary health centers, 1 secondary health facility, and 3 traditional health facilities were studied making a total of 24 health facilities. The director or manager of the health facility and the most senior health worker were interviewed in each selected health facility. 

### 2.6. Method of Data Collection

Interviewer-administered questionnaires were used to obtain information from the heads of households. A household is skipped if on the second visit, the head of household is not found. 

Questionnaires were administered to the director or manager or the most senior staff in charge of physiotherapy services in the health facility on the day of visit. Observation and inspection of available manpower, equipment, and treatment protocols for physiotherapy services at each of the health facility was also carried out. These were carried out by the researcher assisted by eleven paid physiotherapists. There was no Physiotherapy service or Physiotherapist found in all. 

### 2.7. Method of Data Analysis

Data was organized bearing in mind the objectives of the study. Tables and figures were used to highlight relationship between variables. Data analysis was done using SPSS 19.0 computer software. Descriptive statistics (frequency, percentage) were utilized. Confidence limit was set at 95%. 

### 2.8. Ethical Considerations

Ethical approval for the study was obtained from the research ethics committee of the University of Nigeria Teaching Hospital Enugu, Nigeria. Permission to carry out the study was obtained from the “Igwes” (Community heads) of the study areas, while verbal consent was obtained from the heads of the households in the communities of the study area.

## 3. Results

No Physiotherapy service or Physiotherapist was found in all the health facilities and communities.

**Figure 1:**
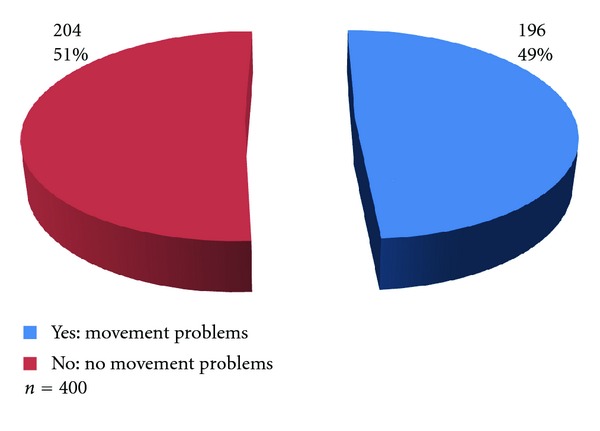
Prevalence of movement/functional problems in households.

Results showed that nearly half of all the participants interviewed 196 (49.0%) complained of having some movement/functional problems (Figure 1).

The majority of respondents with movement/functional problems were having spinal problems 75 (18.8%), followed by those having multiple joint problems 51 (12.8%) and lower limb joint problem 43 (10.8%). The least problem was upper limb joint problem 8 (2.0%).

A fifth 79 (19.8%) of the respondents were experiencing these problems occasionally, while 60 (15.0%) were having these problems all the time (Table 2).

The majority (55, 28.1%) of these respondents visited chemist for treatment of their movement/functional problems followed by those who used home remedy 33 (16.8%). Only few visited a tertiary or general hospital 12 (6.1%) each, while 39 (19.9%) sought no therapy for the problem (Table 2).

Of those who sought help for movement/functional problems, a majority of 115 (73.3%) either received drugs, traction, and/or Plaster of Paris (POP) as treatment. Only one person (0.6%) had physiotherapy included in the treatment regimen.

The major reasons for choice of health facility include cheap 66 (33.7%), accessibility 44 (22.4%), and expert care 26 (13.3%) (Table 3).

On the whole, 53 (27.0%) visited a traditional health facility of which the majority 29 (54.7%) received topical herbs as treatment followed by 14 (26.4%) who received traditional bone setting (Table 4).

**Figure 2:**
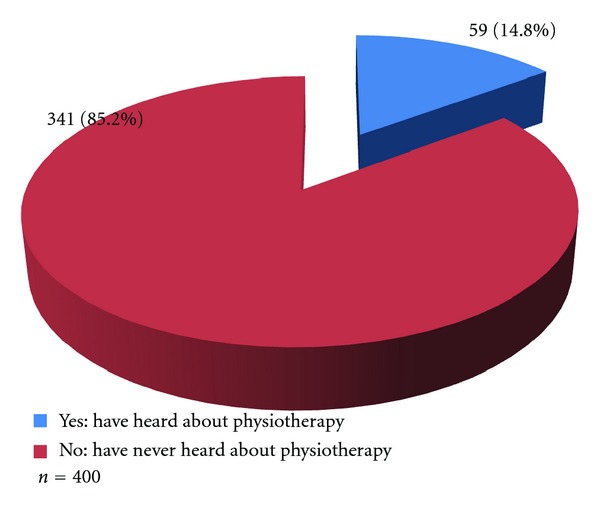
Knowledge of physiotherapy by respondents in the communities.

A majority (341, 85.2%) of the respondents have never heard about physiotherapy (Figure 2).

**Figure 3:**
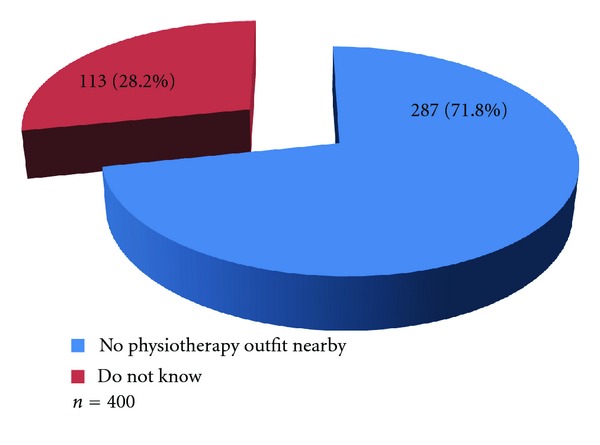
Availability of physiotherapy facilities within the community.

Results show that none of the respondents acknowledged having any physiotherapy outfit nearby (Figure 3).

All the health facilities neither have nor offer physiotherapy services.

Many health workers 10 (41.7%) reported having no qualified personnel for physiotherapy services as their reason for lacking physiotherapy services, while an equal number 10 (41.7%) felt physiotherapy services were not needed in their health facility (Table 5).

**Figure 4:**
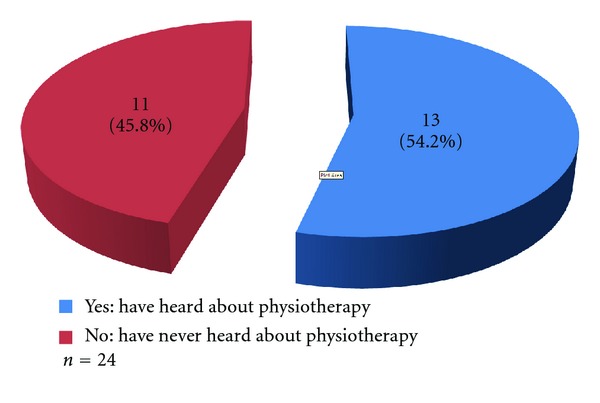
Knowledge of physiotherapy by health workers.

Results show that many of the health workers 11 (45.8%) had never heard about physiotherapy (Figure 4). 

## 4. Discussion

It was discovered that the majority of the respondents having movement/functional problems visited “chemist,” sought no help or used home remedy for their movement/functional problems with majority being treated with drugs, traction and, or Plaster of Paris (POP). Only few visited tertiary health facility, out of which only one person was referred for physiotherapy. This highlights the poor knowledge and referral status for physiotherapy. Their main reasons for choice of health facility were affordability, accessibility, and expert care. This finding is similar to the findings of Vindigni et al. [15] that the main barriers to managing musculoskeletal conditions for rural Aboriginal communities were that majority of the respondents that reported musculoskeletal conditions did not receive treatment or management because they had learned to live with the problem, were unaware of what might help, or found private therapies too expensive [15]. A similar result was given by Akinpelu et al. in 2010, which showed that very few participants with functional problems as a result of musculoskeletal pain in rural communities sought hospital treatment while majority used self-prescribed drugs for pain alleviation [16]. 

Over four-fifths of the respondents in the households had never heard about physiotherapy, and there was no physiotherapy outfit in the community. This result agrees with several other studies supporting that rural communities lack knowledge of physiotherapy and lack physiotherapy services [1, 5, 9, 17–25]. 

A significant number of the respondents eventually visited traditional health facility for their movement/functional problems with majority either receiving topical herbs or traditional bone setting as treatment. The health workers in the study area were mainly primary health care workers though a significant number were Traditional Birth Attendants and traditional healers. Several studies support that traditional health workers significantly contribute to the number of medical complications presenting to hospitals especially in developing nations [26–30]. 

More than half of the health workers encountered movement/functional problems in their facility, which is mainly treated using oral NSAIDs and herbs/traditional bone setting. Very few are referred to tertiary health facility. Many health workers reported having no qualified personnel for physiotherapy and physiotherapy not necessary as their reasons for lacking physiotherapy services in their health facility. Additionally, nearly half (45.8%) reported having no knowledge of physiotherapy. This shows that though there was a high burden of movement/functional deficits especially involving the spine, multiple joints, and muscles in the communities, many people may not be appropriately referred for physiotherapy. Many of the health workers feel they had no need for physiotherapy probably because they did not know the role and scope of physiotherapy and think their treatment options involving mainly the use of oral NSAIDs are optimal. Studies, however, have shown that oral NSAIDs produce short-term relief and are associated with increased risk of gastrointestinal haemorrhage especially when taken for prolonged periods of time [31–36]. Physiotherapy, on the other hand, has been shown to be better in the long term with effects that can be sustained. However, physiotherapy when combined with short-term topical NSAIDs produces the best results [31–36]. This is because the pain relieving effects of the NSAIDs allowed for better exercise tolerance.

The obstacles to receiving optimal physiotherapy in this community were that the people have poor health care seeking behavior. They made their health care choices based on affordability, accessibility, and “expert care” (Table 3). They may have also felt that the movement and functional loss was inevitable, hence some did not seek help nor do anything for their movement/functional loss. Majority of them have no knowledge of physiotherapy, hence may not have the choice of self-referral for Physiotherapy. There were also no physiotherapy services available neither in the communities nor in the health facilities to address the movement and functional problems. Also quite a number of the health workers completely had no knowledge of physiotherapy therefore may not refer patients appropriately. A significant number of the health workers felt they had no need for physiotherapy in their health facilities most probably because they did not know the role and scope of physiotherapy and because few people were presenting to the health facilities with movement/functional problems, a typical “ice-berg” phenomenon.

These obstacles are, however, different for developed countries like Australia, Canada, United Kingdom, and United States of America, where their rural health workers appreciated the need for physiotherapy and understood the role and scope of physiotherapy. The rural residents knew the role and scope of physiotherapy hence could be available for self-referral if optimal physiotherapy services were to be provided [37–39]. 

## 5. Conclusion

The obstacles to obtaining optimal physiotherapy services in this community were unavailability of physiotherapy services, poor knowledge of health workers 11 (45.8%) and the community dwellers 341 (85.2%) of the roles and scope of physiotherapy, poor health care seeking behavior of the community dwellers 50 (25.5%), patronage of traditional health workers 53 (27.0%), and poor referral practices by the health workers 1 (0.6%). 

## Figures and Tables

**Table 1:** 

Ward	Population	Private health facility	Primary health centers	Secondary health facility	Tertiary health facility	Traditional health facility	Total health facilities
Agbani (studied)	20,612	5	3: Ogbeke, Ojiagu and Mgbogodo Health Center	1: Agbani General Hospital	none	1	10
Akegbe Ugwu 1	8,756	1	1: Akegbugwu Health Center	None	None	None	2
Akegbe Ugwu 2 (studied)	8,349	1	1: Our Lady Health of the SickHealth Center	None	None	None	2
Amodu (studied)	9,902	None	1: Amodu Health Center	None	None	1	2
Amurri (studied)	19,385	None	2: Amurri Health Centers 1&2	None	None	1	3
Ndiuno Uwani (studied)	8,632	1	1: Ndiuno Uwani Health Center	None	None	None	2
Obe (studied)	9,635	None	1: Obe Health Center	None	None	None	1
Obinagu Uwani (studied)	12,656	1	1: Obinagu Uwani Health Center	None	None	None	2
Obuno	9,856	None	1: Obuno Health Center	None	None	None	1
Obuoffia	10,141	1	2: Obuoffia and Amangwu Health Centers	None	None	None	3
Ogonogo-ejindiagu	18,306	1	1: Ogonogo-ejindiagu health Center	None	None	None	2
Ogonogo-ejindiuno (studied)	10,848	None	1: Ogonogo-ejindiuno health Center	None	None	None	1
Ozalla	9,311	None	2: Ozalla and Model health Centers	None	1: University of Nigeria Teaching Hospital	None	3
Umueze (studied)	4,108	None	1: Umueze health Center	None	None	None	1

Total	160,497	11	19	1	1	3	35

**Table 2:** Household respondent's movement/functional problems and frequency of occurrence.

Movement/functional problems	Frequency of occurrence			
	All the time	Most times	Occasionally	Total no (%)
	no	no	no	
Spinal problems	14	23	38	75 (18.8)
Multiple joint problems	14	17	20	51 (12.8)
Lower limb joint problem	16	12	15	43 (10.8)
Upper limb joint problem	5	1	2	8 (2.0)
Limb weakness	11	4	4	19 (4.8)
None	—	—	—	204 (51.0)

Total no (%)	60 (15.0)	57 (14.3)	79 (19.8)	400 (100.0)

*n* = 400.

**Table 3:** Management of movement/functional problems at household level.

	Number of respondents	Percentage (%)
First health care provider		
Tertiary hospital	12	6.1
General hospital	12	6.1
Private hospital/clinic	16	8.2
Health center	10	5.1
Chemist	55	28.1
Traditional health facility	18	9.2
Prayer house	1	0.5
Home remedy	33	16.8
No action	39	19.9

Total	196	100.0

Initial treatment received		
Drugs, traction, and/or pop	115	73.3
Herbs and/or traditional bone setting	22	14.0
Hot water and balm	11	7.0
Referral to secondary or tertiary health facility	3	1.9
Massage	2	1.3
Surgery	2	1.3
Prayers	1	0.6
Physiotherapy and others	1	0.6
None	39	19.9

Total	196	100.0

Reasons for choice of facility		
Cheap	66	33.7
Accessibility	44	22.4
Expert care	41	20.9
Referral	26	13.3
Afraid of therapy	19	9.7

Total	196	100.0

*n* = 196.

**Table 4:** Use of traditional health facility for functional problems by households.

	Number of respondents	Percentage (%)
Visit to traditional health facility		
Yes	53	27.0
No	143	73.0

Total	196	100.0

Traditional treatment received		
Topical herbs	29	54.7
Traditional bone setting	14	26.4
Nothing	6	11.3
Oral herbs	2	3.8
Massage	2	3.8
Did not seek traditional treatment	143	73.0

Total	196	100.0

*n* = 196.

**Table 5:** Reasons for lack of physiotherapy services in the 24 health facilities in the study area.

	Number of respondents	Percentage (%)
Why is there no physiotherapy services in your facility		
Lack of trained personnel	10	41.7
Not necessary	10	41.7
Lack resources for equipments	2	8.3
Lack space for such services	2	8.3

Total	24	100.0

*n* = 24.
